# 

**Published:** 1966-07

**Authors:** 


### Contents

| | | Page |
|---|---|---|
| THE EVOLUTION OF LIVER SURGERY ...... | *A. G. Riddell* | 39 |
| ADVANCES IN THE MANAGEMENT OF Rh HAEMOLYTIC DISEASE ...................... | *Geoffrey H. Tovey,* M.D., F.C. PATH. | 46 |
| VIRUSES AND CANCER ........................ | *Edward S. Meek* | 52 |
| "BROOK'S FAMILY HERBAL" .................................. | *A. P. Radford,* M.B., CH.B., D.C.H. | 55 |

